# BSN-ESC: A Big–Small Network-Based Environmental Sound Classification Method for AIoT Applications

**DOI:** 10.3390/s23156767

**Published:** 2023-07-28

**Authors:** Lujie Peng, Junyu Yang, Longke Yan, Zhiyi Chen, Jianbiao Xiao, Liang Zhou, Jun Zhou

**Affiliations:** Department of Internet of Things Engineering, School of Information and Communication Engineering, University of Electronic Science and Technology of China, Chengdu 611731, China; 202011012345@std.uestc.edu.cn (L.P.); 202121010231@std.uestc.edu.cn (J.Y.); 2020020912024@std.uestc.edu.cn (L.Y.); 202221011608@std.uestc.edu.cn (Z.C.); jianbiao_x@std.uestc.edu.cn (J.X.); zlzl@uestc.edu.cn (L.Z.)

**Keywords:** environmental sound classification, low computational complexity, neural network, AIoT

## Abstract

In recent years, environmental sound classification (ESC) has prevailed in many artificial intelligence Internet of Things (AIoT) applications, as environmental sound contains a wealth of information that can be used to detect particular events. However, existing ESC methods have high computational complexity and are not suitable for deployment on AIoT devices with constrained computing resources. Therefore, it is of great importance to propose a model with both high classification accuracy and low computational complexity. In this work, a new ESC method named BSN-ESC is proposed, including a big–small network-based ESC model that can assess the classification difficulty level and adaptively activate a big or small network for classification as well as a pre-classification processing technique with logmel spectrogram refining, which prevents distortion in the frequency-domain characteristics of the sound clip at the joint part of two adjacent sound clips. With the proposed methods, the computational complexity is significantly reduced, while the classification accuracy is still high. The proposed BSN-ESC model is implemented on both CPU and FPGA to evaluate its performance on both PC and embedded systems with the dataset ESC-50, which is the most commonly used dataset. The proposed BSN-ESC model achieves the lowest computational complexity with the number of floating-point operations (FLOPs) of only 0.123G, which represents a reduction of up to 2309 times in computational complexity compared with state-of-the-art methods while delivering a high classification accuracy of 89.25%. This work can achieve the realization of ESC being applied to AIoT devices with constrained computational resources.

## 1. Introduction

In recent years, environmental sound classification (ESC) has attracted increasing research attention and is widely used in many artificial intelligence Internet of Things (AIoT) applications, such as audio surveillance [1,2], wild animal monitoring [3], and smart homes [4]. For example, in audio surveillance applications, ESC technology can be used for the detection of security events, such as gun shots and screaming. In smart home applications, ESC technology can be used to alert parents when their babies cry [5]. ESC usually consists of two parts: feature extraction and classification. For feature extraction, there are some acoustic features that are often used, including the raw sound wave, frequency spectrum [6], gammatone filterbank cepstral coefficient (GFCC) [7], mel-frequency cepstral coefficient (MFCC) [8], and logmel spectrogram [9]. Among them, the logmel spectrogram is used in our work. Firstly, it is not a good choice to use the raw wave of sound as the input feature due to the large data amount with redundant information. Compared with the MFCC, GFCC, and logmel spectrogram, the frequency spectrum lacks information in the time domain. Compared with the MFCC and GFCC, the logmel is realized using almost the same method as the MFCC/GFCC, with the only difference being that it eliminates the discrete cosine transform (DCT). Both the MFCC and the GFCC perform the DCT to keep information on the spectral envelope, but it discards information on pitch. In fields such as speech recognition or keyword spotting, which focus on the human voice, the glottal vibration (related to pitch) is redundant information, so DCT can be performed to discard information on pitch. However, in the ESC, information on pitch is also important for the classification of sound events, and, thus, the DCT cannot be used. Eliminating DCT can also significantly reduce computational complexity. Therefore, in recent years, many state-of-the-art works have used logmel as the input feature. We have also followed this trend.

For classification, the extracted features are classified into different environmental sounds with a traditional machine learning-based or deep-learning-based classifier. Machine learning methods have been widely adopted in the ESC in the past. Some widely used machine learning methods include decision tress (DT) in [10], K-nearest neighbors (KNN), and support vector machine (SVM) in [11]. However, these methods cannot extract and utilize effective feature information well, so the classification accuracy is limited [12]. To address this problem, deep learning methods, such as convolutional neural network (CNN) and recurrent neural network (RNN), have been used to improve the classification accuracy by learning the effective features from the training data automatically. However, a major problem with neural network-based deep learning methods is that they usually involve a large number of floating-point operations (FLOPs), resulting in a large amount of processing time. This is a big challenge for the ESC being applied on AIoT devices with constrained computational resources. In this work, a big–small network-based ESC method (named BSN-ESC) has been proposed to significantly reduce the amount of computation while achieving high classification accuracy. The main contributions of this work include the following:

- A big–small network-based ESC model is proposed to reduce the amount of computation while achieving high classification accuracy by assessing the classification difficulty level of the input audio clip and adaptively activating a big or small network for classification.
- A pre-classification processing technique with logmel spectrogram refining is proposed to further improve the classification accuracy.
- The proposed BSN-ESC model is implemented on FPGA hardware for the evaluation of processing time of AIoT devices.
- The proposed BSN-ESC method is evaluated on the commonly used ESC50 dataset. Compared with several state-of-the-art methods, the number of FLOPs is significantly reduced (up to 2309 times) while achieving a high classification accuracy of 89.25%.
- The rest of this paper is organized as follows. Section 2 reviews the related work. Section 3 presents the details of the proposed BSN-ESC method. Section 4 presents the hardware implementation of the BSN-ESC on the FPGA. Section 5 shows and discusses the experimental results, and Section 6 concludes the paper.

## 2. Related Work

In the past, various traditional machine learning methods have been adopted for ESC. For example, KNN and SVM have been used in [11], in which the outputs of both KNN and SVM were converted into probabilistic scores, and then the two scores were fused into a frame score. The classification accuracy obtained using the combination of KNN and SVM is higher than that of using either of them alone. In addition, DT was adopted in [10], in which the decision on each segment of the audio clip was averaged by the proposed algorithm, with the decisions of the past segments based on the DT.

However, the limited classification accuracy was the common drawback of these traditional machine learning-based methods. The features used in these methods were usually extracted manually through feature engineering, which was not only time consuming but also inefficient in extracting effective features for achieving high accuracy. Actually, the accuracy heavily depended on the designer’s experience. 

In recent years, the deep-learning-based methods based on the end-to-end neural network have been proposed to improve the classification accuracy of ESC by automatically learning the effective features from audio clips. One of the earliest deep learning models for ESC was proposed in [13], named PiczakCNN, where a 2-D convolutional neural network (CNN) was used to analyze the logmel features with two convolution layers and two fully connected layers. Later, the image recognition network models, such as GoogleNet and AlexNet [14], have been adopted for ESC as the logmel spectrograms extracted from audio clips could also be regarded as images. Later, researchers began to apply techniques, such as the data augmentation, transfer learning, and attention mechanism, to ESC in order to improve the classification accuracy. The effects of data augmentation, such as time stretching, pitching shifting, and background noise on the performance of ESC, were studied in [15,16]. A multi-stream CNN with temporal attention has been proposed in [17] to address the robustness issue cross different ESC tasks. Based on this method, a combined temporal and channel attention mechanism has been proposed in [18] to enhance the representative power of CNN by generating complementary information, which obtained higher classification accuracy. Refs. [19,20,21,22] used modified network models from the Residual Networks (ResNet) together with other innovative methods, which improved the classification accuracy. Among them, Ref. [19,20] used modified network models from the ResNet50 while [21,22] used modified network models from the ResNet18. [19] showed that transfer learning with models pre-trained on ImageNet has proven to be successful for ESC, and [20] proposed a new data augmentation technique of triplicating and random-masking the input logmel spectrograms. A recognition method based on multi-feature parameters and time-frequency attention module has been proposed in [21] to extract the attention weight of the input feature spectrogram and to reduce the interference coming from the background noise, and the irrelevant frequency band and [22] constructs a feature generative replay model for ESC that can imitate the human memory process without forgetting old knowledge when learning new knowledge to achieve the fusion of the new and old task information. A method based on Mel-spectrogram separation and long-distance self-calibration CNN has been proposed in [23], and it could retain the original information of the feature map while extracting new features to protect the effective information of the output layer in model training to improve the classification accuracy.

Compared with the traditional machine learning-based ESC methods, the aforementioned deep-learning-based ESC methods based on end-to-end neural network have achieved higher classification accuracy, but they have resulted in a significant increase in the number of FLOPs, which has led to large processing times and power consumption. Therefore, people began to investigate how to reduce the number of FLOPs in the deep learning ESC methods. A one-dimensional (1D) convolutional neural network was proposed in [24] to use two separable 1D convolution filters factorized from two-dimensional (2D) convolution filters for reducing the computational complexity. In [25], a new convolutional neural network architecture with the widening factor was developed to change the number of parameters and FLOPs of systems, and a method based on an increase in the stride size (from 2 to 4) of few convolutional layers was proposed to improve the inference efficiency of CNN-based systems for ESC tasks. In [26], knowledge distillation strategy, which takes advantage of the redundancy in the neural network, was proposed to reduce the number of FLOPs. In [27,28], the standard convolutions were replaced with the depthwise separable convolution (DSC) or hybrid convolution, which combines the traditional convolution and DSC at different stages to further reduce the number of FLOPs. The above methods reduce the number of FLOPs to some extent, but the reduction of computational complexity is always accompanied by the loss of classification accuracy. In addition, the computational complexity after reduction is still not enough to fit on AIoT devices with heavily constrained computational resources and power budgets.

## 3. Proposed ESC Method

This section presents our proposed ESC method, including a big–small network- based ESC model (named BSN-ESC) for reducing the amount of the computation while achieving high classification accuracy, as well as a pre-classification processing technique with logmel spectrogram refining (PPTLSR) for further improving the classification accuracy.

**Figure 1:**
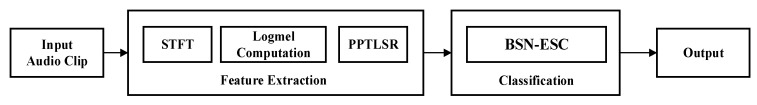
Overall architecture of the proposed ESC method.

Figure 1 shows the overall architecture of the proposed ESC method. It mainly consists of two parts: the feature extraction part and the classification part. In the feature extraction part, the short-time Fourier Transform (STFT) is first performed on the input audio clip (usually several-second duration) and the sampling time is 23.2 ms per frame, with a sampling number of 1024 and a sampling rate at 44.1 kHz. There is also a 50% overlap between two neighboring frames. This is followed by the logmel computation, where a number of frames after STFT goes through the mel filter bank with 128 triangular band-pass filters and the log computation module. After that, the output of logmel computation is processed by the PPTLSR module to obtain three logmel spectrograms of size (128 × 256). The logmel spectrograms are then fed into the BSN-ESC model one-by-one for computation. The details of each module are described as follows.

### 3.1. Proposed BSN-ESC Model

The existing end-to-end neural network models usually contain only one big network. However, the classification difficulty level of input audio clips varies from one to another. In other words, some audio clips are difficult to classify and need a big network to achieve high accuracy, while some audio clips are easy to classify and need only a small network to achieve high accuracy. Therefore, using a big network for all the audio clips is a kind of waste in term of the amount of computation. Based on this observation and analysis, we propose the BSN-ESC model, which can assess the classification difficulty level and adaptively activate a big or a small network to significantly reduce the amount of computation while maintaining high accuracy.

**Figure 2:**
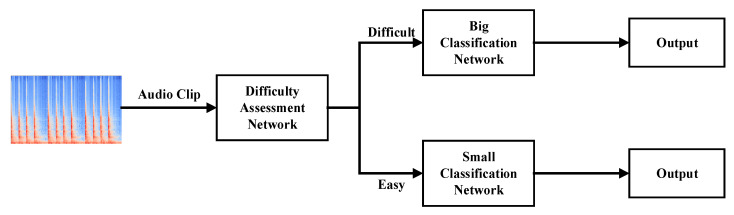
Proposed BSN-ESC model.

The BSN-ESC model mainly consists of three parts, as shown in Figure 2: the difficulty level assessment network, the big classification network, and the small classification network. For an input audio clip, the assessment network is used to assess its classification difficulty level by training the network to classify the input audio clips into two classes (i.e., Difficult or Easy). Then, for the ‘Difficult’ audio clip, the big classification network will be activated to process it. For the ‘Easy’ audio clip, the small classification network will be activated to process it. By extensive experiments, we have observed that most of the audio clips can be classified into ‘Easy’. Therefore, the small classification network will be activated much more frequently than the big network, leading to large saving of the computation while maintaining high accuracy.

**Figure 3:**
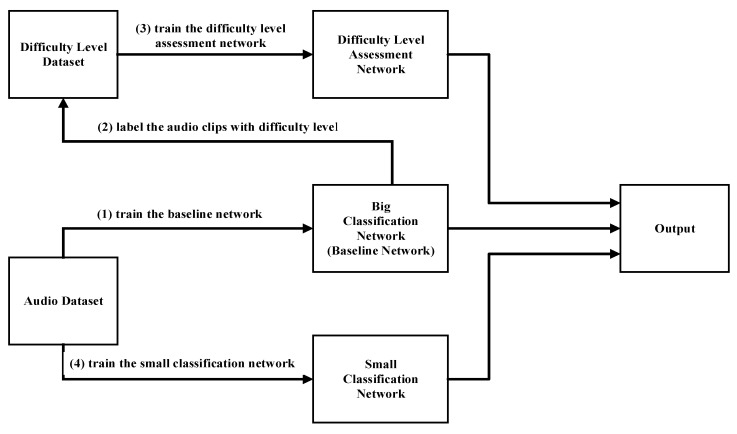
Proposed BSN-ESC model.

The training process of the difficulty level assessment network and the big and small classification network is shown in Figure 3. Firstly, we use the network shown in Table 1 as our baseline network. The audio clips in the training dataset are fed into the baseline network to obtain the accuracy result for each environmental sound class. Then, a pre-defined accuracy threshold is used to divide the environmental sound classes into the ‘Difficult’ class and ‘Easy’ class. For example, there are five environmental sound classes A, B, C, D, and E, and their accuracies are 0.89, 0.92, 0.72, 0.85, and 0.97. If the accuracy threshold is set to 0.88, all the audio clips belonging to A, B, and E are labelled as ‘Easy’ classes because the accuracies of these classes are higher than the threshold, and all the audio clips belonging to C and D are labelled as ‘Difficult’ classes because the accuracies of these classes are lower than the threshold. All the labelled audio clips are then used to train the difficulty level assessment network. After that, the big and small classification network are also trained independently using the audio clips in the training dataset. In our experiment, for simplification, we directly used the baseline network as the big classification network. The detailed structures of the three network are shown in Table 1. Following previous work [24], the big and small network contain both normal convolution layers and depthwise separable convolution layers to achieve relatively low complexity with high accuracy, while the assessment network is much smaller as it is only used for 2-class classification. During the testing, the assessment network will assess the difficulty level of each of the input audio clips in the test dataset and adaptively activate the big or small classification network.

### 3.2. Proposed PPTLSR Technique

**Figure 4:**
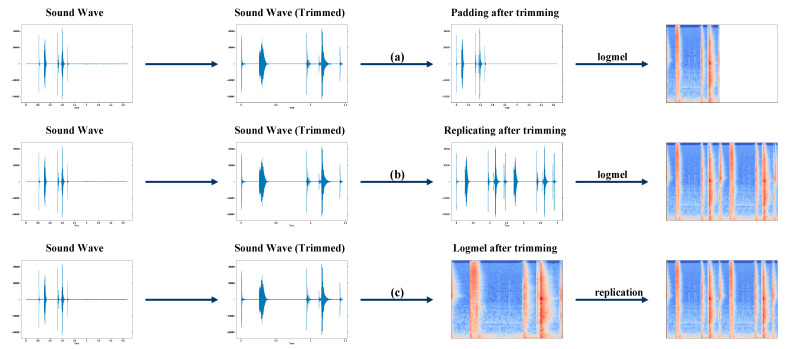
The proposed PPTLSR technique vs. existing pre-processing techniques in ESC: (a) Padding after trimming; (b) Replicating after trimming; (c) Logmel after trimming.

The audio clip often contains silent parts with background noise at the start or the end, which do not carry useful information but affect the classification accuracy. Therefore, trimming the silent parts at the start or the end of a sound clip is a common pre-processing technique in ESC to improve the accuracy. However, the lengths of the sound clips change after trimming the silent parts. As the neural network model can only take sound clips with uniform length, padding is required after the trimming. There are usually two ways of padding, as shown in Figure 4a,b. One way is to pad zeros to the trimmed sound clip to the uniform length. Another way is to pad the trimmed sound clip by repeating itself until the uniform length is reached. After the padding, the logmel spectrogram of the sound clip is generated and fed to the neural network for classification. Previous work showed that the latter method can achieve higher accuracy than the former [18]. However, we have found an issue in the latter method. The frequency-domain characteristics of the sound clip could be distorted at the joint part of two adjacent sound clips because of frame-sliding operation in the logmel spectrogram generation process. This will affect the classification accuracy. In this work, instead of padding in the time domain, we proposed the PPTLSR technique, in which the padding is performed after the logmel spectrogram generation, as shown in Figure 4c. The logmel spectrogram of the sound clip is generated first, and then the logmel spectrogram is repeated until the uniform length is reached. In this way, the distortion in the frequency domain can be prevented, which helps improve the classification accuracy, as can be seen in the section of experimental results.

## 4. FPGA Based BSN-ESC Hardware Implementation

**Figure 5:**
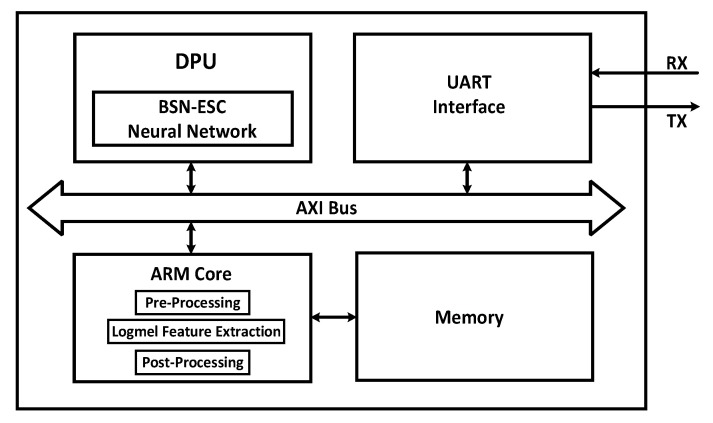
System architecture of the FPGA-DPU.

To evaluate its performance on PC and an embedded system, the proposed BSN-ESC model has been implemented on both CPU and FPGA, respectively. For the FPGA implementation, the BSN-ESC model has been implemented on a Xilinx ZCU104 FPGA board with a Deep-Learning Processor Unit (DPU). The DPU is an IP core provided by Xilinx FPGA for accelerating neural network. As shown in Figure 5, the DPU is used to accelerate the BSN-ESC neural network, and the ARM core is used to implement the other parts, including the pre-processing, logmel feature extraction, and post-processing. The AXI bus is used for data transfer between the ARM and the DPU. An UART interface is used to send the audio clip data from the dataset on the laptop to the FPGA, and send the classification results from the FPGA back to the laptop. 

**Figure 6:**
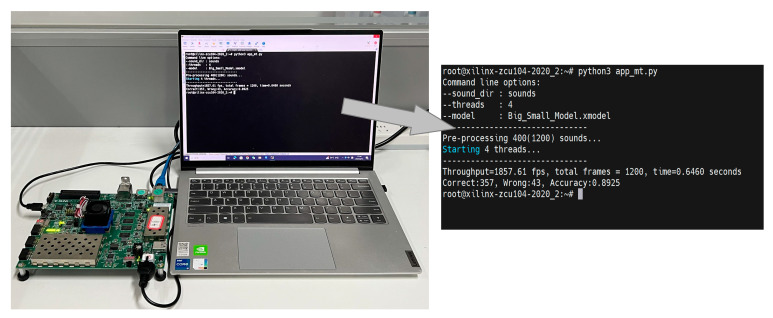
FPGA test setup.

The Xilinx Vitis AI software is used to deploy the BSN-ESC neural network on the DPU. It can finetune, quantify, and compile the trained models of neural networks into instructions. The instruction and the weights are then loaded into the DPU for the acceleration of the neural network. The extracted logmel features are sent to the DPU through the AXI bus by the ARM core, and the classification results are read back by the ARM core after the neural network computation is completed. Figure 6 shows the FPGA test setup and the screenshot on PC. As shown in the screenshot, the proposed method is implemented on ZCU104 FPGA board and the classification accuracy is 89.25%.

## 5. Experiments and Discussion

### 5.1. Dataset

The commonly used public dataset ESC-50 [29] is used to evaluate the performance of the proposed BSN-ESC method. The ESC-50 dataset is a labelled set of 2000 audio clips made for the benchmark methods of environmental sound classification, containing various types of sounds, such as “animals sounds”, “natural soundscapes and water sounds”, “human, non-speech sounds”, “interior/domestic sounds”, and “exterior/urban noises”. Each audio clip is 5 s long with a sampling frequency of 44.1 kHz, and the 2000 audio clips are divided into 50 classes (40 examples per class).

### 5.2. Pre-Processing and Data Augmentation

In the logmel pre-processing, each frame consists of 1024 sample points with 50% overlap with the next frame. The number of frames X of a trimmed sound after logmel module can be represented as in:

X = (t × 44100)/512 − 1,(1)

where t is the time length of the trimmed sound. The obtained logmel spectrogram with X frames is then replicated several times until the number of frames reaches 512. 

**Figure 7:**
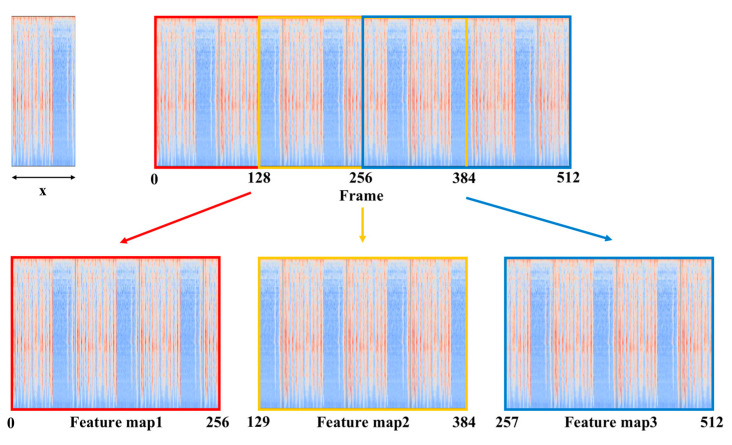
Process of logmel spectrogram splitting.

In order to make efficient use of the limited training data to improve the accuracy, the logmel spectrogram with 512 frames is split into three new logmel spectrograms, each containing 256 frames with 50% overlap. The size of the new logmel spectrogram is 128 × 256. The process is shown in Figure 7. 

To further improve the accuracy, data augmentation methods, such as time masking [30] and mix-up [31], are adopted. By randomly setting the values of t frames in a spectrogram to 0 and mixing two masked spectrograms with different proportional coefficients, the new spectrograms are generated and added for data augmentation.

### 5.3. Training and Testing Method

In the training phase, each logmel spectrogram after splitting is sent to the neural network for the weight training, while in testing phase 3, logmel spectrograms after splitting are sent to the neural network sequentially and their results are averaged to obtain a classification result. N-fold cross-validation is adopted in the experiment, and the value of N is 5. In other words, the ratio of the number of training samples and test samples is 4:1. We used the pytorch library to train the proposed model and used stochastic gradient descent with Nesterov momentum of 0.9 to optimize the model parameters by minimizing the cross-entropy loss of a minibatch, which consists of 64 spectrograms. A total of 80 epochs are set for the training of the model. The learning rate is initialized to 0.01, and for every 30 epochs, it is reduced by 10 times.

### 5.4. Results and Analysis

**Figure 8:**
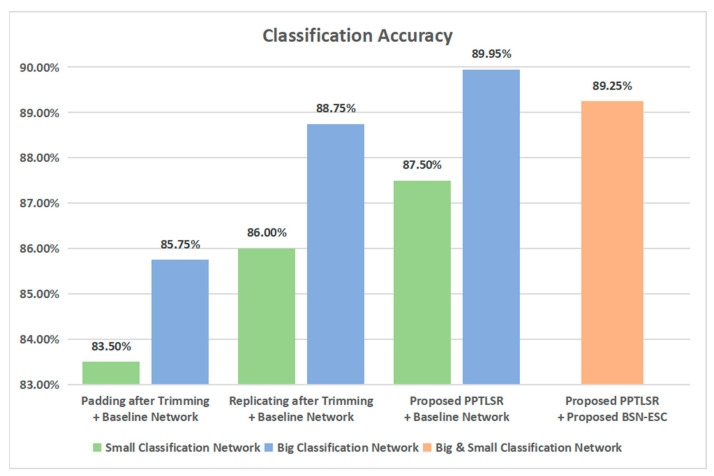
Classification accuracy of the proposed method.

Figure 8 and Table 2 show the classification accuracy and processing time of different methods. As shown in Figure 8, the proposed PPTLSR is applied on both the big classification network and the small classification network individually and jointly. For the performance of the PPTLSR on the big classification network, the accuracy of “Padding after Trimming + Baseline Network” is 85.75%. The accuracy increases to 88.5% when “Replicating after Trimming + Baseline Network” is adopted. With the “Proposed PPTLSR + Baseline Network”, the accuracy increases to 89.95%, and for the performance of the PPTLSR on the small classification network, the accuracy is 83.5% when using “Padding after Trimming” and increases to 86% when using “Replicating after Trimming”. After adopting the proposed PPTLSR, the accuracy increases to 87.5%. With “Proposed PPTLSR + Proposed BSN-ESC Network”, which means the PPTLSR is applied on the big and small network jointly, the accuracy slightly decreases to 89.25% compared with that of the big classification network, but this reduces the computational complexity by 3.4 times and reduces the processing time by 2.1 times, as shown in Table 2. During the experiment, the big classification network has been activated 62 times and the small classification network has been activated 338 times. Please note that in Table 2, the “proposed BSN-ESC Network” includes the big and small classification network and the assessment network.

Table 3 compares the proposed method with the state-of-the-art work. As shown in the table, the number of FLOPs of the compared work range from 0.249 G to 284.06 G, while the proposed method has only 0.123 G FLOPS, which is the lowest, and 2309 times less than [17]. This number of FLOPs (0.123 G) of the proposed method includes that of the assessment network and the big and small classification network. According to the difficulty level given by the assessment network, 15.75% of the audio clips are labelled as ‘Difficult’ class and activated the big classification network, while 84.25% of the audio clips are labelled as ‘Easy’ class and activated the small classification network. Thus, the total number of FLOPs is obtained by the formula shown below:

15.4% × 0.417 G (big) + 84.6% × 0.068 G (small) + 1.35 M (assessment) = 0.123 G,(2)

The classification accuracy of the proposed model is 89.25%, which is the third highest and only lower than [19,20]. However, the number of FLOPS of the proposed method is 2103 times less than [19] and 1491 times less than [20]. This shows that the proposed method is able to achieve significant reduction on the computational complexity while maintaining high accuracy. It is suitable for ESC on AIoT devices with constrained computational resources.

## 6. Conclusions

In this work, a new ESC method named BSN-ESC is proposed to address the high computational complexity of the existing ESC methods. The proposed BSN-ESC model includes a big–small network-based ESC model that can assess the classification difficulty level and adaptively activate a big or a small network for classification as well as a pre-classification processing technique with logmel spectrogram refining that prevents the distortion in the frequency-domain characteristics of the sound clip at the joint part of two adjacent sound clips. The proposed BSN-ESC model achieves the lowest computational complexity with the number of floating-point operations (FLOPs) of only 0.123G, which represents a reduction up to 2309 times in computational complexity compared withe state-of-the-art methods. In the meanwhile, the proposed BSN-ESC model delivers a high classification accuracy of 89.25%, which is the third highest, but the number of FLOPs of the other two methods are 2103 times and 1491 times higher than ours. In addition, with our proposed logmel spectrogram refining technique, compared with the baseline network, the computational complexity is reduced by 3.4 times and the processing time is reduced by 2.1 times. This work can achieve the realization of ESC being applied to AIoT devices with constrained computational resources. In the future, we will implement the proposed lightweight algorithm on hardware, building a practical application in the real-time environment.

## Figures and Tables

**Table 1:** Detailed Structure of the Three Networks.

Difficulty Level Assessment Nework	Big Classification Network	Small Classification Network
1 × 5 Standard Conv1	3 × 5 × 32 Standard Conv1	3 × 5 × 32 Separable Conv1
3 × 3 Maxpooling1	3 × 5 × 32 Separable Conv2	4 × 3 Maxpooling1
(easy/hard) FC-sigmoid	4 × 3 Maxpooling1	3 × 1 × 64 Standard Conv2
	3 × 1 × 64 Standard Conv3	4 × 1 Maxpooling2
	3 × 1 × 64 Separable Conv4	1 × 5 × 128 Separable Conv3
	4 × 1 Maxpooling2	1 × 3 Maxpooling3
	1 × 5 × 128 Standard Conv5	3 × 3 × 256 Separable Conv4
	1 × 5 × 128 Standard Conv6	2 × 2 Maxpooling4
	1 × 3 Maxpooling3	(# of classes) FC-softmax
	(# of classes) FC-softmax	

**Table 2:** Processing Time and Computational Complexity of the Proposed Method.

Methods	Processing Time		Computational
			Complexity
	CPU	FPGA	(No. of FLOPs)
Proposed PPTLSR +Baseline Network	30.66 s	3.36 s	0.417 G
Proposed PPTLSR +Proposed BSN-ESC Network	14.66 s	2.35 s	0.123 G

**Table 3:** Comparison of the Proposed Method with the Existing Methods.

Methods	Classification Accuracy	Computational Complexity
	ESC-50 Dataset	No. of FLOPs
Multi-Stream CNN [17] 2019	84.0%	284.060 G
ZhangCNN [18] 2019	86.5%	0.485 G
SoundCLR [19] 2021	92.9%	258.74 G
ESResNet [20] 2021	91.5%	183.36 G
LGTFB [24] 2020	86.2%	0.812 G
LCSED [27] 2022	83.0%	2.640 G
ULSED [28] 2022	88.3%	0.249 G
Multi-Feature CNN [21] 2022	89.0%	1.82 G
ERANN [25] 2022	89.2%	10.01 G
LSCNet [23] 2022	88.0%	0.65 G
FGR-ES [22] 2023	82.08%	1.82 G
Ours	89.25%	0.123 G
